# Proton Triggered Colorimetric and Fluorescence Response of a Novel Quinoxaline Compromising a Donor-Acceptor System

**DOI:** 10.3390/s18103433

**Published:** 2018-10-12

**Authors:** Yogesh W. More, Sachin D. Padghan, Rajesh S. Bhosale, Rajendra P. Pawar, Avinash L. Puyad, Sidhanath V. Bhosale, Sheshanath V. Bhosale

**Affiliations:** 1 Polymers and Functional Material Division, CSIR-Indian Institute of Chemical Technology, Hyderabad 500 007, India; yogeshwmore@gmail.com (Y.W.M.); padghansachu@gmail.com (S.D.P.); bhosale@iict.res.in (S.V.B.); 2 Department of Chemistry, Indrashil University, Kadi, Mehsana 382740, India; 3 Department of Chemistry, Deogiri College, Aurangabad 431005, India; rajendrapawar61@gmail.com; 4 School of Chemical Sciences, Swami Ramanand Teerth Marathwada University, Nanded 436106, India; avinashlpuyad@gmail.com; 5 Department of Chemistry, Goa University, Taleigao Plateau Goa 403206, India

**Keywords:** colorimetric, fluorescent, proton sensor, quinoxaline, reversible sensor

## Abstract

Quinoxaline-based novel acid-responsive probe **Q1** was designed on the basis of a conjugated donor-acceptor (D-A) subunit. **Q1** shows colorimetric and fluorometric changes through protonation and deprotonation in dichloromethane. With the addition of the trifluoroacetic acid (TFA), UV-vis absorption spectral changes in peak intensity of **Q1** was observed. Moreover, the appearance of a new peaks at 284 nm 434 nm in absorption spectra with the addition of TFA indicating protonation of quinoxaline nitrogen and form **Q1.H^+^** and **Q1.2H^+^**. The emission spectra display appearance of new emission peak at 515 nm. The optical property variations were supported by time resolved fluorescence studies. The energy band gap was calculated by employing cyclic voltammetry and density functional calculations. Upon addition of triethylamine (TEA) the fluorescence emission spectral changes of **Q1** are found to be reversible. **Q1** shows color changes from blue to green in basic and acidic medium, respectively. The paper strip test was developed for making **Q1** a colorimetric and fluorometric indicator.

## 1. Introduction

In live cells the intracellular and extracellular pH are changed by variations in physiological processes. It is well reported that inflammation and tumor growth may be associated with an acidic pH in living systems [1], making pH sensors are useful for cellular analysis or diagnosis. In recent year researchers have developed several pH sensors to determine glucose [2], urea [3], penicillin [4], acetylcholine [5], creatinine [2], halogenated hydrocarbons [6] and organophosphorus pesticides [7]. A literature search reveals that many pH sensors have been employed in numerous fields such as environmental monitoring, bioprocessing and biomedical applications [8,9,10,11,12,13]. pH is measured by using large number of analytical tools such as glass electrodes and photometric methods and are many are commercialized. Though these methods are well established, they have several disadvantages such as the need for frequent calibration of pH electrodes, corrosion caused by alkaline solutions or fluoride ions and the electrodes’ susceptibility towards interference leading to limited usefulness [14]. To overcome these disadvantages scientists have directed their efforts towards the development of optical sensors [15] particularly fiber-optic pH sensors [16,17,18,19]. In recent years fluorescence techniques have been employed to measure pH with high sensitivity [20,21,22]. Fluorescence changes have been observed due to the protonated and deprotonated state of a chromophore, which in turn displays change in color of a dye [23]. Such colorimetric and fluorescent pH sensors are appropriate for the living samples because of their high signal-to-noise ratio and also their physical stability. For real-world applications, simple design and easy synthesis of a fluorescent chromophore as sensor is an important field of research. A literature search revealed that quinoxaline derivatives have been employed for various applications such as organic photovoltaics, organic light emitting diodes (OLEDs) and dye sensitized solar cells, etc. [24,25,26,27].

Quinoxaline chromophores have been also used as an anion sensor, colorimetric and ratiometric cation sensor [28,29,30]. Duke and co-workers reported a bisquinoxaline amidothiourea-based anion sensor [31]. Zhang et al. reported preparation of a quinoxaline-based thin film and its use as a solid-state pH sensor for organic acids, amine and amino groups [32]. Quinoxaline derivatives are also used as a pH sensor [33,34,35,36]. Therefore, we have chosen a malononitrile-functionalized quinoxaline derivative. In this manuscript, we report the synthesis and sensing properties of quinoxaline chromophore **Q1**. The proton sensing properties were monitored through optical, UV-vis and fluorescence spectroscopic changes. On the basis of our findings, finally we employed the system to prepare paper strip and tested it for the detection of protons via a colorimetric method.

## 2. Experimental Details

### 2.1. Materials and Methods

All the reactions were carried out under nitrogen atmosphere, solvents used for the reactions were dried using appropriate drying agents. Solvents for column chromatography used as received without further purification and all other reagents were used as supplied by commercial vendors without further purification, unless indicated. Anhydrous DCM was prepared for cyclic voltammetry using an Innovative Pure Solve solvent purification, typically, DCM kept overnight on calcium chloride. Then decant and reflux over phosphorous pentoxide for 1 h, distilled and collected over activated molecular sieves (4A). Thin layer chromatography (TLC) was performed aluminum coated silica gel (40–60 µm, F254) and visualized either using UV light (254 and 365 nm) or iodine indicator. Compounds were purified by column chromatography on 40-60 silica gel. ^1^H-NMR spectra were recorded at 400 MHz and the following abbreviations are used to explain multiplicities: d = doublet, m = multiplet, dd = doublet of doublets. ^13^C-NMR spectra were recorded at 100 MHz, as indicated. Both ^1^H and ^13^C calibrated using residual non-deuterated solvent as an internal reference and are reported in parts per million (δ) relative to tetramethylsilane (δ = 0). Melting points were measured using a digital melting point apparatus. IR-Spectra were recorded using a Nexus 670 spectrometer (Thermo Nicolet, Boston, MA, USA) using non-hygroscopic KBr pellets.

### 2.2. Synthesis

The quinoxaline **Q1** derivative was prepared in two steps. Step-by-step synthesis details and full characterisation data can be seen in the Supplementary Information.

### 2.3. UV–vis Measurements

UV–vis absorption experiments were performed in a quartz cell with a 1 cm path length. An aliquot of the stock solution of **Q1** (0.2 mL, conc. = 10^−3^ M in dichloromethane) mixed with additional 3 mL dichloromethane and after allowing to equilibrate for 2 h then the absorption spectra was measured.

### 2.4. Fluorescence Measurements

Fluorescence spectra were measured on a RF-6000 spectrofluorophotometer (Shimadzu, Nakagyo-ku, Kyoto, Japan) using a quartz cell with a 1 cm path length (λ_ex_ = 350 nm).

### 2.5. Time Resolved Fluorescence Studies

The fluorescence lifetime measurements were carried out by the time correlated single photoncounting technique (TCSPC) with a micro channel plate photomultiplier tube (MCPPMT) as the detector and a nanosecond laser as the excitation source (model 5000 U, IBH, Glasgow, UK).

### 2.6. Cyclic Voltametry Measurement

These measurements were carried out on a BASI Epsilon EcSoftware system (Autolab Instrument, West Lafayette, IN, USA) in a 3-electrode single compartment cell with Pt disc working electrode (Ø = 0.5 mm), a platinum wire counter electrode (Ø = 0.5 mm) and a silver wire pseudo reference electrode, all these cells and electrodes are custom made. The reduction potentials were referred to SCE using ferrocene (Fc) as internal reference (E1/2 Fc SCE = +0.48 V) after the measurements.

### 2.7. Theoretical Calculations

The theoretical calculations were performed by using B3LYP and basis set 6-311+G(d,p) method.

### 2.8. Paper Strip Experiments

Paper test strip was prepared by using Wattman filter paper 44. The prepared strip from dichloromethane solution of **Q1** (1 mM) is exposed to TFA and TEA vapours. The naked eye color change is noted.

## 3. Results and Discussion

### 3.1. Synthesis and Characterization

**Scheme 1:**
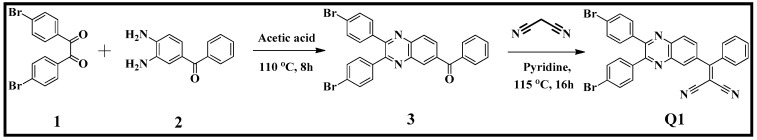
Synthesis of 2-((2,3-bis(4-bromophenyl)quinoxalin-6yl)(phenyl)methylene) malononitrile (**Q1**).

The quinoxaline **Q1** derivative was synthesized in two-steps as shown in Scheme 1. In the first step, 1,2-bis(4-bromophenyl)ethane-1,2-dione (**1**) was treated with the (3,4-diaminophenyl)-(phenyl)methanone (**2**) in acetic acid at 110 °C for 8 h to yield the (2,3-bis(4-bromophenyl)quinoxalin-6-yl)(phenyl)methanone derivative **3**. Compound **3** was further reacted with malononitrile in pyridine at 115 °C for 16 h to yield the desired 2-((2,3-bis(4-bromophenyl)quinoxalin-6-yl)-(phenyl)methylene)malononitrile (**Q1**). The obtained product **Q1** was characterized by FT-IR, ^1^H- and ^13^C-NMR and ESI mass spectral techniques. In the FT-IR spectra of **Q1** the characteristic peak for the nitrile functional group appeared at 2224 cm^−1^. **Q1** showed very good solubility in CHCl_3_, dichloromethane and THF.

### 3.2. Colorimetric Response

**Figure 1:**
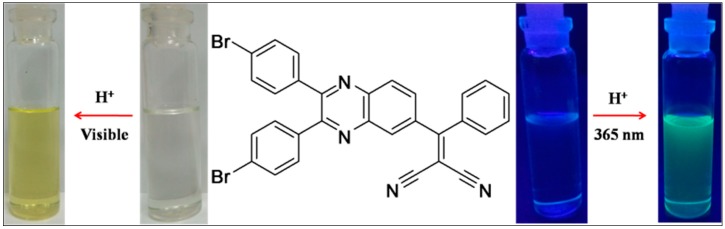
Photograph of the color changes of **Q1** (dichloromethane solution) by treatment with TFA; (**a**) under visible light; (**b**) under UV light of 365 nm.

A naked-eye experiment was recorded in the dichloromethane solution by the addition of 50 equivalents of the TFA to the receptor **Q1** solution (Figure 1). The receptor solution showed a color change in color from colorless to yellow under visible light and blue to fern green under UV light (365 nm), respectively. These observations suggest that **Q1** showed sensitivity towards TFA via protonation of ring nitrogen atoms.

### 3.3. UV-vis and Emission Spectral Changes

**Figure 2:**
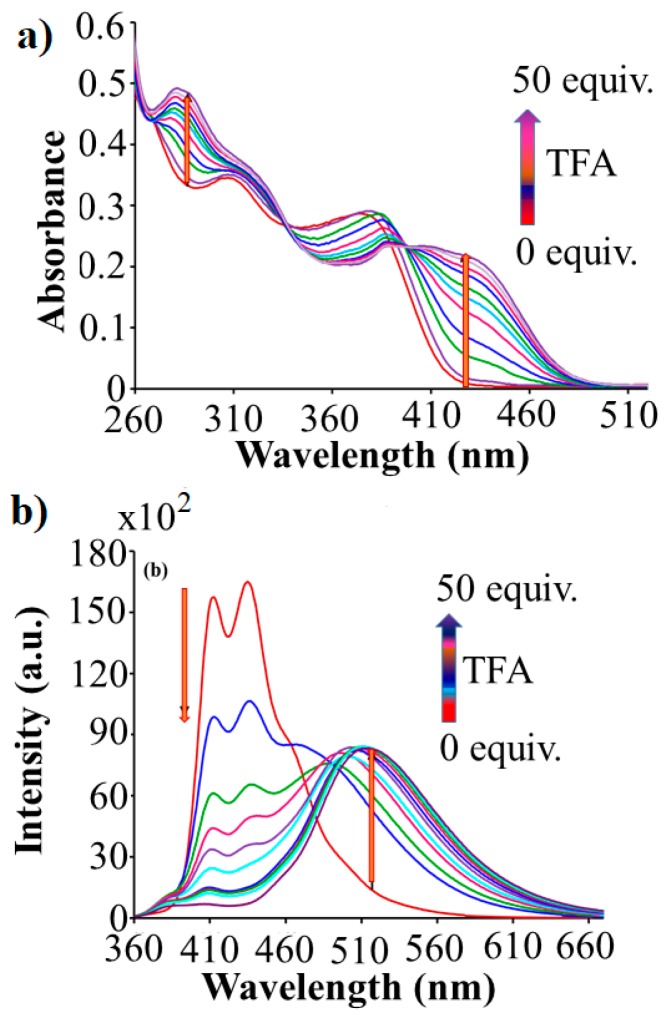
(**a**) Absorption spectra of compound **Q1** (10 µM) and (**b**) Fluorescence emission spectra of compound **Q1** (10 µM, λ_exc_ = 350 nm) in dichloromethane.

The UV-vis absorption spectra of **Q1** recorded in dichloromethane solutions with increasing added TFA are displayed in Figure 2a. **Q1** showed two absorption peaks centred at 310 nm and 377 nm. Interestingly, upon addition of TFA to the dichloromethane solution of **Q1**, absorption bands at 310 nm and 377 nm showed a significant red shift to 319 nm with an increase in intensity and at 389 nm with a decrease in intensity, respectively. With increasing TFA concentration in **Q1** solution, a new absorption bands appeared at 284 nm and 434 nm. The broad and intense absorption band at 284 nm 434 nm is ascribed to the electronic transition in the π-molecular system and clearly indicates formation of two protonated states [37] such as monoprotonation **Q1.H^+^** and diprotonation **Q1.2H^+^** of the receptor **Q1**.

Fluorescence spectroscopy was used to investigate proton detecting ability of **Q1** in dichloromethane. The emission spectra of **Q1** in dichloromethane showed two sharp peaks centred at 413 nm and 436 nm upon excitation at 350 nm (Figure 2b). The titration of **Q1** with TFA (0-50 equiv.) resulted in a decrease in the band intensity at 413 nm and at 436 nm along with the appearance of new emission band at 515 nm. We presume that the decrease in peak intensity at 413 nm and 436 nm may be due to formation of monoprotonated state of the **Q1** as **Q1.H^+^**, which in turn leads to form diprotonation state **Q1.2H^+^** with the incremental addition of TFA, indicating by appearance of new emission peak at 515 nm.

### 3.4. Time Correlated Single Photon Counting (TCSPC) Studies

**Figure 3:**
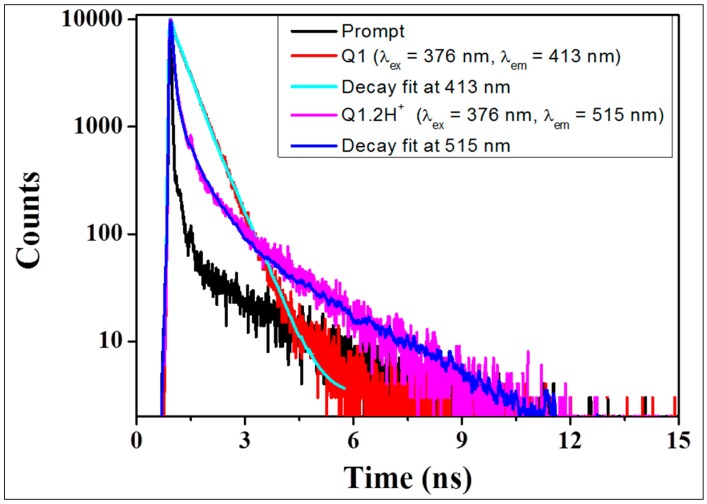
TCSPC decay histograms and fitted bi-exponential decay functions for **Q1** and **Q1.2H^+^** in dichloromethane.

The fluorescence decay time displays the dynamic picture of the obtained fluorescence, which is independent of concentration of solution. Time correlated single photon counting (TCSPC) method used to study the kinetics of the emission via optical excitation of the samples, individual photon arrival time and their detection. TCSPC of **Q1** and **Q1.2H^+^** in dichloromethane with excitation at 376 nm and emissions were recorded at 413 nm and 515 nm, respectively (Figure 3). Decay profiles of **Q1** and **Q1.2H^+^** were fitted with bi-exponential function. In dichloromethane solutions **Q1**, the fluorescence lifetime of components were 0.10 ns (7.70%) and 1.19 ns (92.30%). Fluorescence lifetime of components for protonated state **Q1.2H^+^**were 0.17 ns (65.82%) and 1.46 ns (34.12%). For deprotonated state of **Q1** the percentage weight of shorter component (0.10 ns) was less than the longer component (1.19 ns). Whereas, for protonated state **Q1.2H^+^** the percentage weight of shorter component (0.17 ns) was higher as compared to the longer component (1.46 ns) (Table 1). Weight percentage differences of shorter and longer components of **Q1.2H^+^**with **Q1** possibly arise from protonation of the nitrogen atoms in quinoxaline ring of probe **Q1** lead aggregates in solution.

### 3.5. Electrochemistry and DFT Calculation Studies

**Figure 4:**
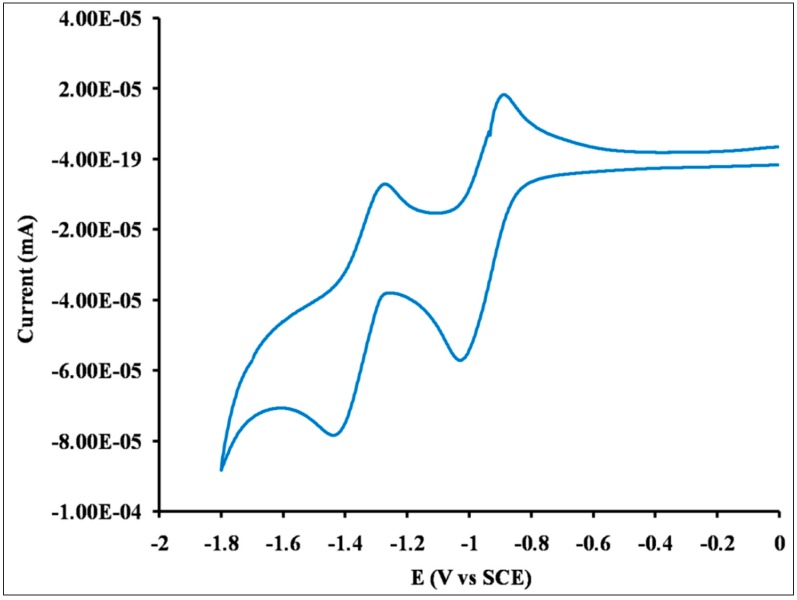
Cyclic voltammetry curve of **Q1** in dichloromethane (electrolyte: 0.1 M *n*Bu_4_NPF_6_, scanning speed: 100 mV·s^−1^).

The CV curve of **Q1** (Figure 4) in dichloromethane showed two well resolved reversible reduction waves. The position of energy levels was estimated from CV curve and UV-vis absorption spectra of **Q1**. The CV curve of **Q1** exhibit first reduction onset potential of −0.852 V and the LUMO energy level can be estimated to be −3.848 eV. The low lying LUMO energy level suggests that **Q1** is good potential n-type candidate. The absorption onset (Figure 2a) of **Q1** is of 424 nm and the estimated optical band gap to be 2.92 eV. Based on the results, optical band gap of LUMO of **Q1** and the HOMO energy level is estimated to be −6.777 eV.

**Figure 5:**
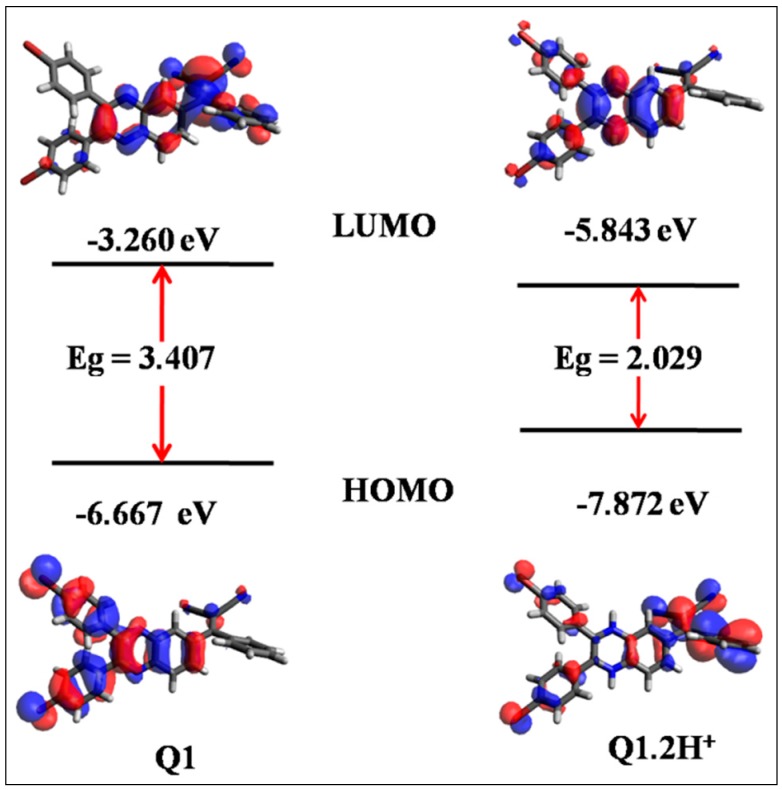
Theoretically calculated frontier orbital of **Q1** and **Q1.2H^+^** calculated by using DFTB3LYP/6-311+G(d,p) basis set.

To investigate the structure-electronic property relationship of **Q1** employed DFT calculations using the Gaussian 09 ab initio/DFT quantum chemical simulation package [38]. Optimization of receptor **Q1** and its protonated form **Q1.2H^+^** using hybrid density functional B3LYP model and 6-311+G(d,p)basis set was carried out in dichloromethane. The effect on geometries of **Q1** and **Q1.2H^+^**are comprised by means of the polarizable continuum model (PCM). The frequency calculations are also carried out to ensure structures to be real. Based upon molecular geometry of the receptor **Q1** and protonated form of receptor **Q1.2H^+^**, the frontier molecular orbitals (FMO) are calculated at the same level of theory and generated using Avogadro (Figure 5) [39,40]. Thus, the electronic distributions in HOMO and LUMO for the receptor **Q1** and **Q1.2H^+^**are as shown in Figure 5. For **Q1**, the electronic density in HOMO is delocalized over the bromophenyl and central region of the quinoxaline subunit. In the LUMO level, electron density is localized on the quinoxaline and malononitrile subunits. In the molecule **Q1**, the electronic distributions of HOMO and LUMO level are not well separated, suggesting that the electronic transitions are not pure charge transfer but may be treated as π-π transitions. Furthermore, we have investigated protonated form of the quinoxaline receptor to investigate the protonation effect on optical properties by means of optimizing the geometry. The changes in electron distribution in the HOMO of **Q1.2H^+^**were observed and are located on bromophenyl and malononitrile subunits i.e. away from quinoxaline subunit in the receptor. In LUMO, the electron distribution was observed over the complete molecule, suggesting that protonation on the **Q1** receptor increases the electron-accepting ability and it lowering of HOMO and LUMO energy gaps from 3.40 eV to 2.02 eV. For the protonated state **Q1.2H^+^**aromatic conjugation mostly stabilize the LUMO and led red-shift **(**higher wavelength) emission spectra (Figure 2b). The calculated band gap using DFT results shows the trend in accordance with the experimental results and does not give the exact value due to delocalization error [41]. This indicate the accurate calculation of band gap using DFT is one of the challenge.

### 3.6. Reversibility of the Receptor

**Figure 6:**
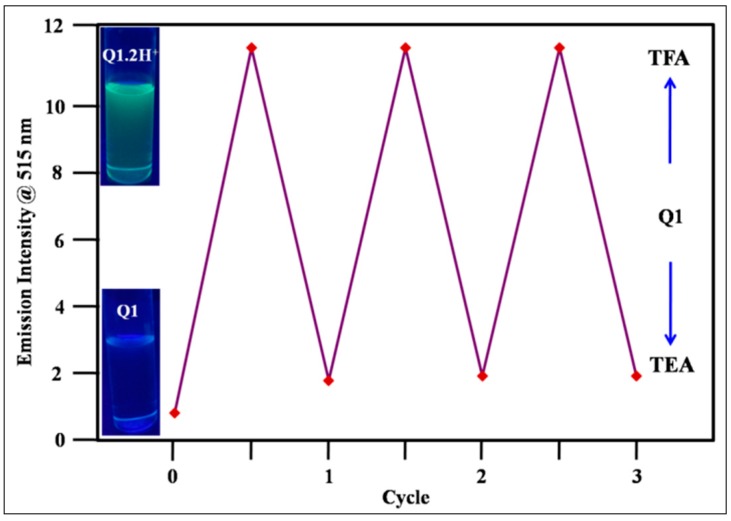
The fluorescence intensity at 515 nm of **Q1** upon consecutive addition of TFA and TEA solution for up to 3 cycles in dichloromethane (Inset at left: color change in **Q1** with protonation and deprotonation under UV light of 365 nm).

The reversible fluorescence emission spectrum of **Q1** was monitored by addition of acid (TFA) and base (TEA). The reversibility has been investigated by recording the fluorescence emission intensity changes at 515 nm in dichloromethane with respect to a change in the acidic and basic conditions through addition of TFA and TEA, respectively (Figure 6). The reversible behaviour of the receptor **Q1** is displayed in Figure 3. For the receptor **Q1**, the reversibility between the protonation and deprotonation state has been observed up to three cycles without any intensity variation. Thus, we believe that the probe may be employed for real time monitoring of protonation and deprotonation in solution and may applicable to use in biological sciences.

### 3.7. Reversibility of the Receptor Proved by Test Strip

**Figure 7:**
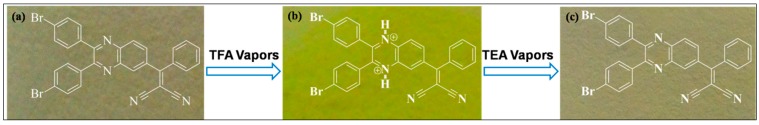
Photograph of test paper strip with **Q1** for reversible proton sensing experiment. (**a**) none (only **Q1**); (**b**) upon exposure with TFA vapours and (**c**) exposure with pure TEA vapours.

Herein, we report an efficient strategy for the prepartion of a proton indicator paper strip using receptor **Q1** (Figure 7). The strip was prepared by immersing Wattman filter paper 44 in the dichloromethane solution of **Q1** (1 mM). The obtained strip was first treated with TFA vapours, and observed that the color of strip changed from cream to yellow. The change in the strip color is almost the same as that in solution. Furthermore, when the yellow strip was treated with vapours of TEA, it was observed that reappearance of the original strip color. It is notable that the color change of strip in film form is very similar to that in dichloromethane solution. Thus, the proton sensitivity of the test strip in dichloromethane was highly reversible, without the leaching of chromophore. These results indicate that the proton response of **Q1** strip was robust and highly reversible.

## 4. Conclusions

In summary, we have synthesized a new quinoxaline-based **Q1** sensor. The receptor Q1 undergoes protonation in the presence of TFA, which can be seen clearly by a change in color from colorless to yellow under visible light and blue to fern green under UV light (365 nm), respectively. The addition of TFA displays efficient changes in UV-vis absorption and fluorescent emission spectra. The optical property variations were also supported by TCSPC studies and the energy band gap was calculated by employing cyclic voltammetry and DFT. Importantly, the receptor can be reused many times, as protonated Q1 can be easily deprotonate upon addition of TEA and is easy to monitor by the naked eye as such color changes from blue to green in basic and vice-versa in acidic medium, respectively. These results clearly suggested protonation of **Q1** in the presence of TFA and deprotonate upon addition of TEA. The strong proton capture capability of **Q1**, allowing reversible fluorescence switching in basic and acidic medium and the emission color changes from blue to green. Furthermore, the paper strip test was developed for making **Q1** as colorimetric and fluorometric indicator for real world applications. Thus, the quinoxaline-based chromophore can be used as a colorimetric indicator for reversible protonation and deprotonation in dichloromethane.

## Figures and Tables

**Table 1:** Fluorescence life time of **Q1** and **Q1.2H^+^** in dichloromethane.

Sample	τ_1_ (ns)	Contribution (%)	τ_2_ (ns)	Contribution (%)
**Q1** (λ_emi_: 413 nm)	0.107	7.70	1.195	92.30
**Q1.**2H**^+^** (λ_emi_: 515 nm)	0.176	65.82	1.466	34.18

## Supplementary Materials

The following are available online at http://www.mdpi.com/1424-8220/18/10/3433/s1. Data SI detail synthesis on intermediate as well as Q1 and FT-IR, ^1^H-NMR, ^13^C-NMR and mass spectra can be found in ESI.
