# Supplementary material for: Proton Triggered Colorimetric and Fluorescence Response of a Novel Quinoxaline Compromising a Donor-Acceptor System
Source: Sensors (Basel). 2018 Oct 12;18(10):3433. doi: 10.3390/s18103433 (PMC6210483; doi:10.3390/s18103433)
Supplement: Supplementary file 1 [file sensors-18-03433-s001.pdf]

# Supplementary Information

# Proton Triggered Colorimetric and Fluorescence Response of a Novel Quinoxaline Compromising A Donor-Acceptor System

**Yogesh W. More [1], Sachin D. Padghan [1], Rajesh S. Bhosale [1,2,*], Rajendra P. Pawar [3], Avinash L. Puyad [4], Sidhanath V. Bhosale [1] and Sheshanath V. Bhosale [5,*]**

[1] Polymers and Functional Material Division, CSIR-Indian Institute of Chemical Technology, Hyderabad 500 007, India; yogeshwmore@gmail.com (Y.W.M.); padghansachu@gmail.com (S.D.P.); bhosale@iict.res.in (S.V.B.)
[2] Department of Chemistry, Indrashil University, Kadi, Mehsana 382740, India;
[3] Department of Chemistry, Deogiri College, Aurangabad 431005, India rajendrapawar61@gmail.com (R.P.P.)
[4] School of Chemical Sciences, Swami Ramanand Teerth Marathwada University, Nanded 436106, India; avinashlpuyad@gmail.com (A.L.P.)
[5] Department of Chemistry, Goa University, Taleigao Plateau Goa 403206, India
* Correspondence: bhosaleo4@gmail.com (R.S.B.); svbhosale@unigoa.ac.in (S.V.B.)

Received: 14 August 2018; Accepted: 8 October 2018; Published: date

## Experimental Procedures

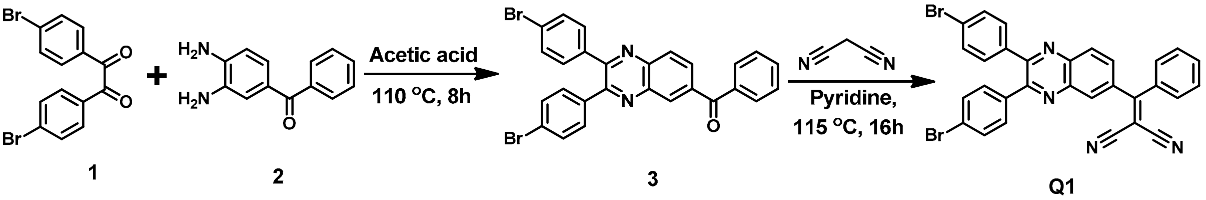


**Scheme S1** Synthesis of compound **Q1**.

## Synthesis of Compound 3

Synthesis of compound **3** was achieved from 4,4'-dibromobenzil **1** and 3,4-diaminobenzophenone **2** *via* cyclic condensation in acetic acid by following reported procedure in the literature.[1]

## Synthesis of Compound Q1

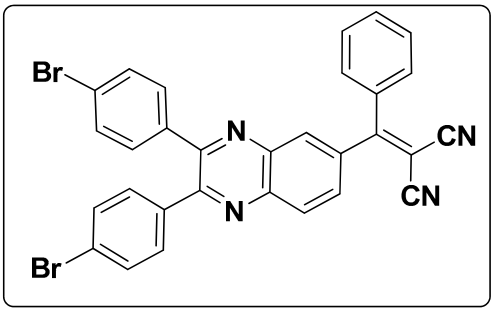


To a 25 ml flask compound **3** (100 mg, 2 mmol), 5 ml pyridine and malononitrile (0.5 ml, 5 mmol) were added. The reaction mixture was heated at 115 $^{\circ}$C for 16 h. Completion of reaction was checked by thin layer chromatography (TLC). The reaction mixture was quenched with ice-cold water and the product was extracted by using EtOAc further washed with 0.1 N HCL solution and brine solution respectively to afford crude solid. The crude product was purified by column chromatography using $CH_2Cl_2$/*n*-hexane mixture as an eluent and yielded pale yellow solid compound **2** (86 mg, 79%). M.p. = 228-230 °C; FT-IR (KBr, $^{-}$ v cm$^{1}$): 538, 593, 707, 766, 825, 976, 1009, 1072, 1182, 1338, 1391, 1443, 1535, 1588, 2224, 2924 and 3439. $^{1}$H NMR ($CDCl_3$, 400 MHz) δ: 7.35 (m, 4H), 7.52 (m, 8H), 7.62 (m, 1H), 7.79 (dd, *J* = 8.5, 1H), 8.25 (d, J= 8.9, 2H). $^{13}$C NMR (100 MHz, $CDCl_3$) δ: 83, 113.5, 128.3, 129.1, 129.7, 130.4, 132.5, 132.9, 135.7, 137.1, 138.3, 140.2, 142.5, 155.1 and 173.5. ESI-MS (*m/z %*): 593 (100) $[M+H]^{+}$.

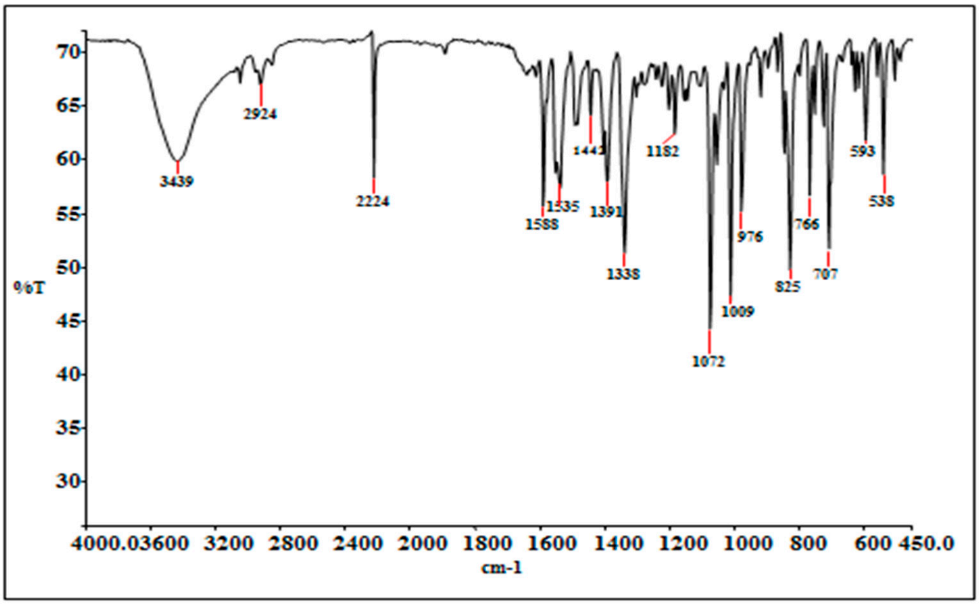


**Figure S1.** FT-IR of compound **Q1**.

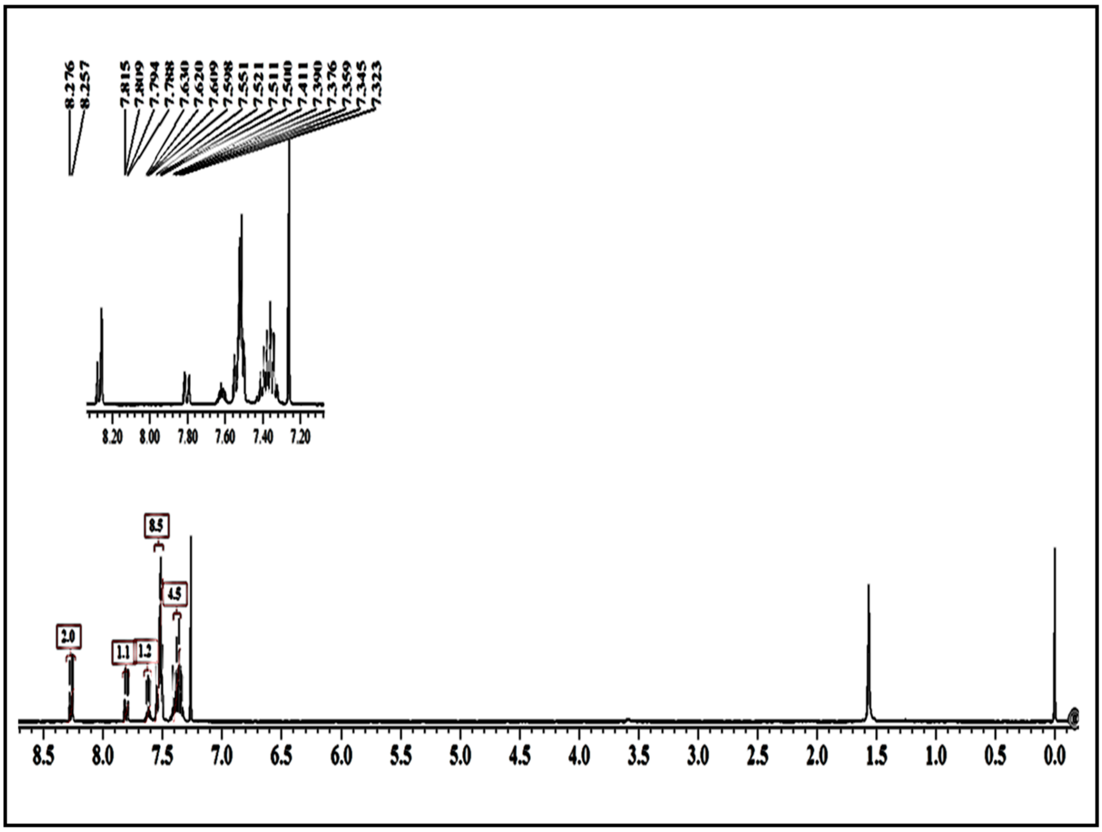


**Figure S2.** $^{1}H$ NMR of compound **Q1**.

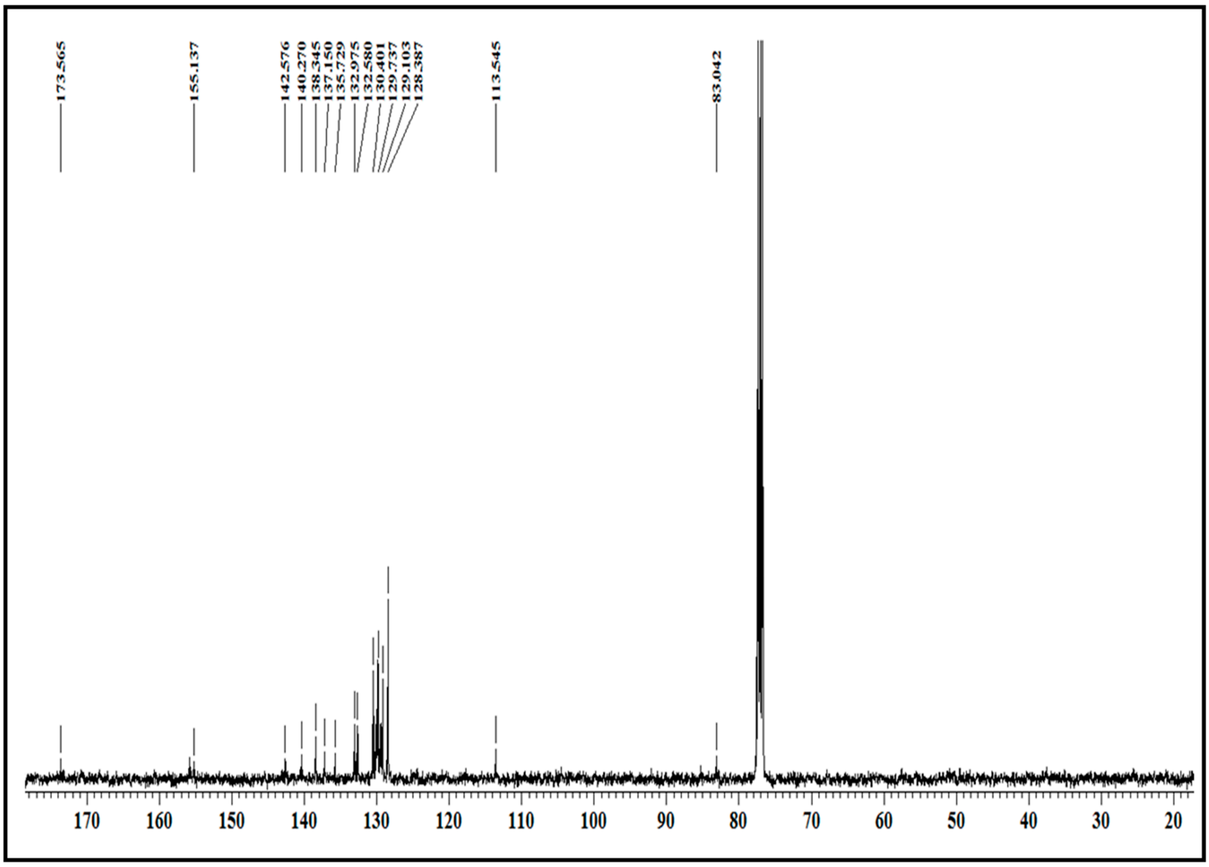


**Fig. S3.** $^{13}$CNMR of compound **Q1**.

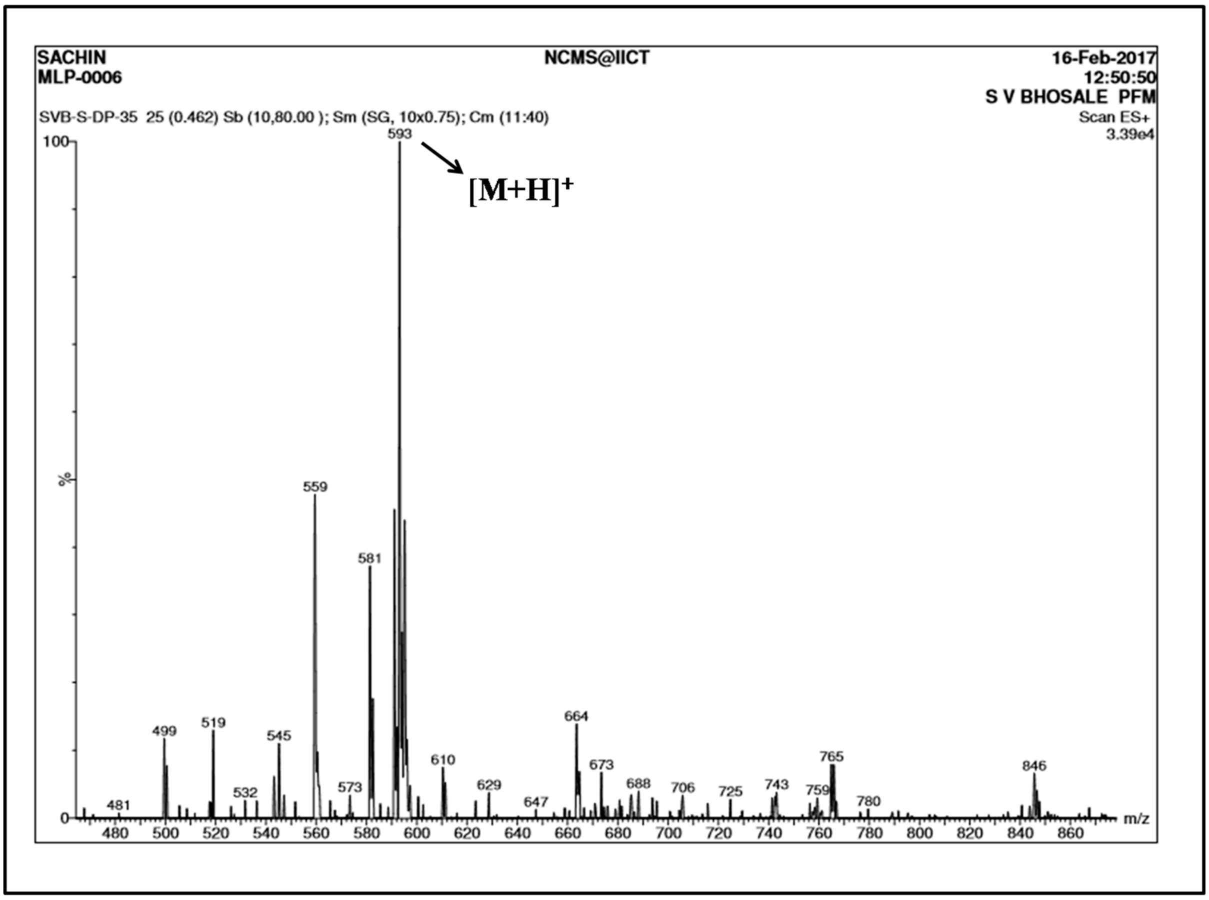


**Fig. S4.** ESI-MS of compound **Q1**.
